# Traditional Chinese Medicine for Liver Cancer Treatment: Network Pharmacology Research

**DOI:** 10.2174/0115680266336478241118065659

**Published:** 2025-01-09

**Authors:** Shihao Zheng, Yixiao Gu, Wenying Qi, Wei Wang, Xiaoke Li, Xiaobin Zao, Size Li, Shaoyu Liu, Tianyu Xue, Yongan Ye, Aimin Liu

**Affiliations:** 1 Department of Spleen and Gastroenterology, Dongzhimen Hospital, Beijing University of Chinese Medicine, Beijing, 100007, China;; 2 Liver Diseases Academy of Traditional Chinese Medicine, Beijing University of Chinese Medicine, Beijing, 100029, China;; 3 Key Laboratory of Chinese Internal Medicine of Ministry of Education and Beijing, Dongzhimen Hospital, Beijing University of Chinese Medicine, Beijing, 100007, China;; 4 School of Traditional Chinese Medicine, Beijing University of Chinese Medicine, Beijing, 100029, China;; 5 Sujiatuo Township Community Health Service Center, Beijing, 100194, China;; 6 Hebei Provincial Hospital of Traditional Chinese Medicine, Shijiazhuang, 050000, China;; 7 Shangzhuang Township Community Health Service Center, Beijing, 100094, China

**Keywords:** Liver cancer, Network pharmacology, Research progress, Traditional Chinese medicine, Targets

## Abstract

**Background:**

As one of the common malignant tumors nowadays, liver cancer has more risk factors for its development and is characterized by a high recurrence rate, high mortality rate, and poor prognosis, which poses a great threat to people's health. The specific efficacy of traditional Chinese medicine is based on clinical practice, which is a high degree of generalization of the characteristics and scope of the clinical effects of prescription medicines and a special form of expression of the medical effects of the human body within the scope of traditional Chinese medicine. Because of its multi-ingredient, multi-target, and multi-pathway characteristics, it has a great advantage in the treatment of liver cancer. Still, at present, its specific molecular mechanism of action has not yet been clarified.

**Aim:**

This study reviews the current status and characteristics of network pharmacology research in the treatment of liver cancer, aiming to provide new ideas and methods for traditional Chinese medicine treatment of the disease.

**Methods:**

This study was searched on the Web of Science and PubMed using keywords, such as “traditional Chinese medicine”, “liver cancer,” and “network pharmacology.” The citation dates of the literature cited in this review are from 2000 to 2024.

**Results:**

The discovery of the key molecular mechanisms of traditional Chinese medicine in the treatment of liver cancer through the network pharmacology approach and the in-depth study of the related signaling pathways are conducive to a more in-depth exploration of traditional Chinese medicine.

**Conclusion:**

Network pharmacology research plays a key role in the treatment and prevention of liver cancer and deserves deeper exploration in the future.

## 1 INTRODUCTION

**Fig. (1):**
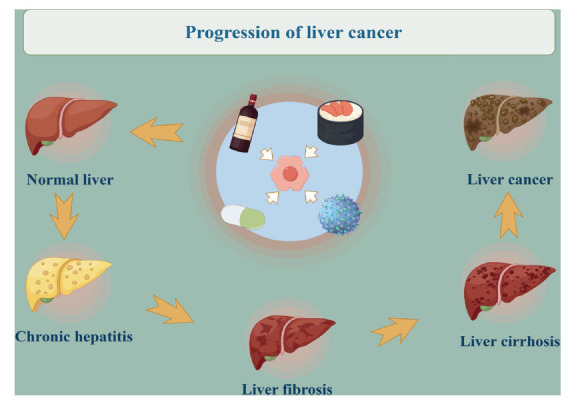
Progression of liver cancer.

Liver cancer is one of the most common malignancies worldwide. Risk factors for liver cancer include hepatitis B virus (HBV), hepatitis C virus (HCV), alcoholic cirrhosis, fatty liver, smoking, diabetes, obesity and dietary factors [1-4]. Specifically, HBV infection is the most important risk factor for the development of liver cancer, accounting for approximately 50% of total cases [5]. It is worth celebrating that the risk of HCV infection has been significantly reduced today as a result of patients achieving a sustained virologic response (SVR) with antiviral drugs [6]. Liver cancer may be triggered by a variety of factors, followed by the development of chronic hepatitis. In severe cases, over time, liver fibrosis or even cirrhosis will occur, ultimately leading to the development of liver cancer. The prognosis is often poor (Fig. **1**). Primary liver cancer is a malignant tumor arising in hepatocytes or intrahepatic bile duct cells, which is characterized by difficult early diagnosis, occult onset, and obscure early symptoms. According to the latest epidemiological data, the global incidence of liver cancer in 2020 is 4.7%, ranking sixth place; the mortality rate is 8.3%, ranking third, and the incidence is growing worldwide. Liver cancer has become one of the serious threats to human life and health diseases [7-9]. The current Western treatment methods for liver cancer include surgical resection, chemotherapy, liver transplantation, radiofrequency ablation, percutaneous hepatic arterial chemoembolization, percutaneous anhydrous ethanol injection, targeted drug therapy and whole-body radiotherapy, but the effect is not satisfactory. Surgical or local treatment has its limitations, which will inevitably damage the normal function of the body. In addition, most patients with primary liver cancer have basic liver disease. With the progression of the disease, liver cancer is found in the middle and late stages, and most patients with abnormal liver function cannot receive radical surgical treatment; the treatment means are very limited, and they can only rely on systemic drug therapy. Moreover, the treatment effect is not very obvious. Specific studies have confirmed that targeted drugs and PD-1 / PD-L1 immune checkpoint inhibitor therapy only produce responses in a portion of the dominant population, covering a small population area [10].

The treatment of liver cancer by traditional Chinese medicine (TCM) emphasizes holistic regulation and treatment. It has the functions of killing liver cancer cells, inhibiting the proliferation of liver cancer cells, improving immunity and, improving patients' physique, *etc*. It can make up for many deficiencies of Western medicine and achieve the purpose of improving symptoms, relieving pain, controlling tumor recurrence and metastasis, prolonging patients' survival period, and improving patients' quality of life. Therefore, more and more scholars at home and abroad pay attention to it. At present, TCM is often used in combination with targeted drugs in the treatment of primary liver cancer, which can have a synergistic effect, reduce its toxic side effects, reverse drug resistance, and increase the clinical benefit rate of patients. The symptoms and signs related to liver cancer were first recorded in the Huang Di Nei Jing, as well as in medical books of all dynasties, and there have been special prescriptions and medicines for the treatment of liver cancer, with remarkable clinical effects, which can alleviate the symptoms of liver cancer patients and improve the therapeutic effect. The prevention and treatment of liver cancer by TCM is an indispensable part of the comprehensive treatment of liver cancer.

**Fig. (2):**
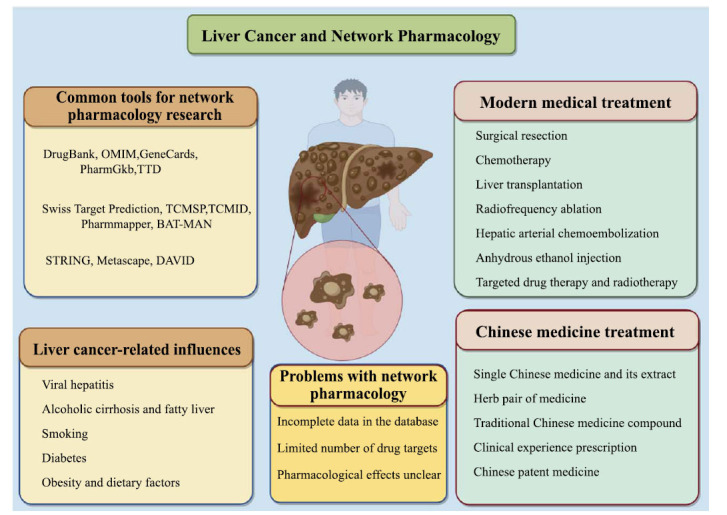
Liver cancer and network pharmacology.

With the rapid development of systems biology, bioinformatics, and a variety of pharmacology, network-based medicine discovery has been recognized as a cost-effective method of medicine development and has been recognized by a large number of scholars [11]. The application of systems biology and other methods to determine the mechanism of action, safety, and pharmacological effects of TCM is of great significance to the research and development of TCM. Therefore, a new interdisciplinary method, network pharmacology, is proposed [12, 13]. The concept of network pharmacology was first systematically proposed and elaborated by British pharmacologist Hopkins in 2007 [14]. With the rapid development of research, network pharmacology has become a new way to study medicine mechanisms and medicine development. Through the research method of network pharmacology, we can directly identify the corresponding targets of diseases and medicines from a large number of data and understand the specific mechanism of medicine action on diseases and related signaling pathways [11, 15-17]. In addition, network pharmacology research methods also include the screening of corresponding targets of active ingredients, target prediction based on medicine algorithms, identification of active ingredients, construction of disease-specific networks, network-based disease gene prediction, and final overall quantitative analysis [18]. In addition, network pharmacology can also construct a network of “ingredient-target-pathway, ” which can describe the complexity and correlation between medicines and diseases from the perspective of a complex network, which coincides with the philosophy of treating diseases in TCM. With the introduction of the concept of network pharmacology, a large number of researches related to network pharmacology of TCM in the treatment of liver diseases and even liver cancer have emerged, which reflects the characteristics of multi-ingredient, multi-target and multi-pathway therapy of TCM in the treatment of liver cancer, giving full play to the therapeutic advantages of TCM. Therefore, this study aims to summarize the current research situation and provide a reference for future research by systematically combing relevant literature (Fig. **2**).

## 2 COMMON TOOLS FOR NETWORK PHARMACOLOGY RESEARCH

### 2.1 Database of Disease Target Screening

First, the DrugBank (https://go.drugbank.com/) database [19], with 2, 358 approved drugs and hundreds of additional information on drug transcriptomics, pharmacometabonomics, and effects on protein expression levels, is known as one of the most comprehensive drug databases available. Second, the OMIM (https://www.omim.org/) database [20], created in the early 1960s, already contains information on more than 16, 000 genes. It is still being updated and is now used by an increasing number of scholars. Third, as one of the most commonly used databases for screening disease targets, the GeneCards database [21] (https://www.genecards.org/) has 150 genes and a unified data center, the network contains the transcription, protein group, genome, and protein targets for clinical disease, is also very comprehensive integrated database in clinical use. Finally, PharmGkb [22] (https://www.pharmgkb.org/) database and TTD [23] (http://db.idrblab.net/ttd/) database are also commonly used. These important databases also provide strong support for the development of network pharmacology.

### 2.2 Database for Screening of Active Ingredients and Target Prediction

First, SwissTargetPrediction [24] (http://swisstargetprediction.ch/) is often used in TCM active ingredients. Target backward prediction of concrete is based on the similarity principle, through a reverse screening to predict possible small molecular target protein. Second, TCMSP [25] (https://old.tcmsp-e.com/tcmsp.php), as one of the most commonly used databases for screening medicine ingredients and targets using network pharmacological methods, has collected nearly 29, 000 active ingredients of hundreds of TCMs. Its corresponding target protein can be screened according to medicine absorption, metabolism, distribution, and other parameters of the active ingredients of TCM and corresponding targets, as well as good stability. Third, the TCM information database [26] (TCMID, http://bidd.group/TCMID/index.html) is a powerful TCM database that integrates the research of medicines, diseases, targets, and prescriptions. It is convenient for relevant researchers to query prescription ingredients and efficacy quickly. It is one of the largest data sets in the field of TCM research and can be used to integrate the relationship between TCM and the diseases it treats, its active ingredients, and its targets.

Finally, other commonly used databases, such as Pharmmapper [27] (http://lilab-ecust.cn/pharmmapper/index.html) database, BAT-MAN [28] (http://bionet.ncpsb.org.cn/batman-tcm/), database, *etc*.

### 2.3 Protein-Protein Interaction (PPI) Network and Database for Bioenrichment Analysis

The biological activities of the body depend on the interaction between biomolecules, and the PPI network can show the interaction of targets at the protein level. The STRING [29] (https://cn.string-db.org/) database integrates all known and predicted protein associations, including physical interactions and functional associations, and covers tens of thousands of protein networks across species. It is one of the most comprehensive data platforms for constructing PPI networks. The Metascape [30] (https://metascape.org/) database is one of the most effective tools for bioenrichment analysis, providing a comprehensive resource for gene list annotation and analysis. It combines interactive group analysis, function enrichment analysis, member search, and gene annotation. However, as the network pharmacology, the most commonly used biological enrichment analysis database, DAVID [31] (https://david.ncifcrf.gov/) database collection the integrated visualization, comments, and found that can effectively provide different tables of gene functional annotation and analysis of microarray data, widely acclaimed by scholars (Table **1**).

## 3 NETWORK PHARMACOLOGY RESEARCH ON LIVER CANCER TREATED BY TRADITIONAL CHINESE MEDICINE

### 3.1 Single Chinese Medicine and Its Extract

#### 3.1.1 Heat-clearing Medicines

*Scutellaria baicalensis* is one of the most common herbs used to clear heat and dry dampness. It has a history of thousands of years. According to TCM, *Scutellaria baicalensis* has a bitter cold flavor and has the function of clearing heat and detoxifying and clearing the fire of upper coke. Modern pharmacology has also proved that the extracts of *Scutellaria baicalensis* flavonoids have extensive pharmacological properties, such as antibacterial, anti-inflammatory, antioxidant, anti-tumor, and liver protection [32]. Wang *et al.* [33] screened out 37 candidate active ingredients of *Scutellaria baicalensis* through network pharmacological analysis and mapped 50 targets corresponding to 29 ingredients of *Scutellaria baicalensis* with targets of liver cancer. Comprehensive compound-target-pathway network analysis found that candidate active ingredients play a role in the treatment of liver cancer by regulating cancer, PI3K-Akt signaling pathway, and other pathways, providing new insights for the mechanism of *Scutellaria baicalensis* therapy of liver cancer and medicine development. Baicalein is one of the key ingredients of *Scutellaria baicalensis*. Ma *et al*. [34] revealed the core modules of PPI through protein-protein interaction (PPI) network construction and topological screening, including two baicalein targets, CDK1 and TP53 and two down-regulated deGs. HSPA1A and HSPA1B, respectively. Finally, it was confirmed that baicalein could exert positive regulatory effects on HCC through the PPI network of CDK1, TP53, HSPA1A, and HSPA1B. When conducting network pharmacological studies on the treatment of liver cancer by *Scutellaria baicalensis,* Gong [35] found that through in-depth analysis of the first signaling pathway and target, only TTR has specific expression in liver tissue and found that TTR may become a potential therapeutic target for liver cancer.

*Hedyotis diffusa Willd* (HDW) has a good effect on clearing heat and detoxifying, and it can also be used in the treatment of humid heat jaundice. Studies have demonstrated that HDW can effectively inhibit the activation of the AKT/mTOR pathway in hepatocellular carcinoma (HCC) cells without causing significant hepatorenal toxicity or weight loss [36]. Wu *et al*. [37] first confirmed the specific therapeutic effect of HDW on liver cancer angiogenesis, then screened out 7 active compounds according to the pharmacokinetic characteristics of HDW, and further found through enrichment analysis and network analysis that these active compounds play an anti-angiogenesis role in liver cancer by acting on multiple targets, mainly involving IL-17 and IL-6. IL-1β, Akt1, and other pathways. This network pharmacological study initially explored the specific therapeutic mechanism of HDW on liver tumor angiogenesis. It provided a new experimental reference for future exploration of its clinical application and pharmacological effects.

As one of the traditional herbal medicines, *Centella asiatica* has a bitter, and cold taste. Based on network pharmacology and lipidomics, Li [38] found that Asiatic acid, a key ingredient of *Centella asiatica*, can inhibit the activation of hepatic stellate cells *in vitro* and effectively relieve liver fibrosis *in vivo*, mainly by participating in the arachidonic acid metabolic pathway, peroxisome proliferator activation receptor signaling pathway and cancer pathway, and ultimately hinder the progress of liver fibrosis. It also prevents liver cancer.

Schistoderma has a long history. Pharmacological studies have provided an important basis for its antitumor, anti-inflammatory, antibacterial, antioxidant, antiviral, and immunomodulatory activities. Through bioinformatics and network pharmacology studies, Yang *et al*. [39] finally obtained 373 potential targets for the treatment of HCC by *Schisandra chinensis*, among which the effective ingredients included quercetin, baicalin, luteolin, baicalin, *etc*., and the core targets included CDK4, CDK1, E2F1, *etc*. Through further enrichment analysis and molecular docking, it was found that the specific mechanism of *scutellaria baicalensis* in treating liver cancer may be to inhibit the expression of core genes by using active ingredients, thus blocking the PI3K-AKT signaling pathway, inhibiting the migration and proliferation of cancer cells, and finally inducing the apoptosis of liver cancer cells.

#### 3.1.2 Medicines Used to Activate Blood Flow

*Salvia miltiorrhiza*, as a well-known medicinal plant, is highly valued in the prevention and treatment of tumors, inhibiting metastasis, proliferation, and angiogenesis, and modulating the tumor microenvironment and immunity [40, 41]. In addition to gynecological diseases, the main clinical applications of *Salvia miltiorrhiza* include early cirrhosis, chronic hepatitis, cerebral ischemia, heart disease, and pulmonary heart disease [42]. Tanshinone IIA (TanIIA), one of the main active ingredients of Danshen, is known to have anti-tumor and circulatory effects. Ma *et al*. [43] found that TanIIA can mediate SMAD7-YAP interaction and inactivate the TGF-β signaling pathway, and finally play a role in inducing apoptosis of liver cancer cells and inhibiting the growth and migration of tumor cells. The final study confirmed that the Tan IIA-SMAD7-YAP regulatory network may become a new idea for the treatment of liver cancer in the future. Luo [44] also discovered the anti-tumor effect of cryptotanshinone (CPT), the active ingredient of *Salvia miltiorrhiza*, on HCC cells through systematic pharmacology and experimental verification and proved the anti-tumor effect of CPT in MHCC97-H and Huh7 cells *in vivo* and *in vitro*. The specific mechanism is to induce autophagy and apoptosis through inhibition of the PI3K/Akt/mTOR signaling pathway and finally play a therapeutic effect.

*Turmeric* has the function of breaking blood, promoting qi, clearing collaterals, and relieving pain. More and more studies have been conducted on the correlation between its main ingredients and liver cancer, which has attracted extensive attention from modern scholars. Curcumol, a main active ingredient extracted from *turmeric* root, has been found to have certain antitumor activity. Huang *et al*. [45] combined bioinformatics and network pharmacology to find that curcumol has 44 effective targets for the treatment of liver cancer. Through further GO analysis and KEGG signaling pathway analysis, they discovered that curcumol increases the presentation of liver cancer-specific proteins mainly by regulating inflammatory response and inducing apoptosis. Finally, it has anti-tumor properties. Yang *et al*. [46] found that curcumin, another active ingredient, also has a good anti-cancer effect. They collected predicted targets of curcumin and liver cancer from multiple databases and finally found that curcumin may alleviate the occurrence and development of liver cancer through the p53 apoptosis pathway and AMPK/ULK1 autophagy pathway. The interaction between apoptosis and autophagy may occur through DRAM and p62. A network pharmacology analysis by Zhao *et al*. [47] showed that GSTM1, TGFB1, TP53, GSTP1, and RB1 are the key genes of curcumin's anti-HCC effect, in which the elevated mRNA expression levels of GSTM1, TP53, and RB1 indicate that the overall survival of HCC is further prolonged. However, elevated TGFB1 mRNA levels suggested a poor prognosis, and this study further broadened the horizons of targeted therapies for HCC. A recent study [48] also found that the combination of Curcumin and 6-shogaol could effectively improve the morphology of HCC cells by acting on the Ras signaling pathway, promote apoptosis in advanced stages, and further impede the G2/M phase cell cycle to inhibit the division and proliferation of cancer cells. In addition, curcumin can effectively inhibit the expression of XRCC4 in HCC cells to promote apoptosis and interfere with the repair process of the nonhomologous DNA terminal link of HCC cells, which ultimately exerts an inhibitory effect on HCC [49]. Although curcumin shows good potential in the prevention and treatment of HCC, most of the studies [50-53] are still in the laboratory stage, and more clinical trials are still needed to validate its efficacy and safety in the future. Meanwhile, improving the delivery efficiency and bioavailability of curcumin is also the focus of future research [54].

*Rubia cordifolia L.* is a medicine commonly used for regulating menstruation in gynecology, which has the unique effect of cooling blood and, removing blood stasis and stopping bleeding. Its widely used rhizome parts have a long history of being used as medicine. Its pharmacological action is very extensive. A pharmacological study [55] confirmed that *Rubia cordifolia L.* Its derived ingredients have anti-tumor, anti-inflammatory, anti-platelet aggregation and anti-oxidation effects and are widely used in traditional medicine. Xiong [56] identified Rubia cordifolia L through databases and literature. Rubia cordifolia L was further constructed by screening and evaluating related ingredients through similarity in medicine and pharmacokinetic characteristics. The ingredient- target-pathway network diagram of anti-HCC was found to inhibit the occurrence and development of HCC through many indirectly acting hepatitis signaling pathways and directly acting tumor-related signaling pathways.

*Purple snowflake* is widely grown in tropical Asia. It also has a good effect on promoting blood circulation and removing blood stasis. The ground portion contains important active ingredients such as plumbagin. As a bioactive compound [57], Plumbagin mainly exists in the Plumbaginaceae family, whose unique anticancer activity has been explored by many scholars. In addition, Plumbagin comes from plants, and its harmless nature is often used to treat malignant tumors, such as liver cancer. Zhou *et al*. [58] discovered the pharmacological targets and molecular mechanisms of Plumbagin against liver cancer by using network pharmacology. The treatment of liver cancer by Plumbagin mainly involves 19 main targets, among which the top five targets are TP53, MAPK1, MAP2K1, RAF1 and CCND1. It was experimentally confirmed that liver cancer sections showed increased expressions of MAPK1 and TP53 in hepatocytes, accompanied by positive clinical imaging results of liver cancer. In HepG2 cells treated with Plumbagin, significantly decreased expressions of TP53 and MAPK1 proteins were observed, accompanied by cell apoptosis and cell arrest. Finally, the inhibitory effect of Plumbagin on liver cancer was confirmed.

#### 3.1.3 Interior-warming Medicine

*Cinnamon* is from the camphor family, camphor is a medium-large tree with bark gray-brown, mostly made of dried bark medicinal materials. Its medicinal properties are relatively hot, and the taste is hot and sweet, such as thirst, dry throat, sore throat, nose bleeding, and other heat symptoms and a variety of acute inflammation should not be taken. *Antrodia cinnamomea* was cinnamomea's main active ingredient with various biological activities, including anti-tumor, liver protection, anti-inflammatory, anti-high fat, antioxidant, antihypertensive, and immunomodulatory activities [59]. *In vivo* and *in vitro* experiments [60], A. cinnamomea dropping pills (ACDPs) significantly decreased the activity of PI3K/AKT signaling pathway and decreased the expression of cycle-related proteins, thereby alleviating the development of liver cancer and promoting the reduction of liver cancer cells. The affinity between the predicted target and its corresponding active ingredient was verified by molecular docking.

*Evodiae Fructus* (EF), as one of the traditional fructus, has a medicinal property of Xin, bitter, hot, has a good cold and pain relief effect, and is more able to stop vomiting, it was born in the flat to 1500 meters above sea level mountain forest or shrubs, mostly in sunny slope, has very good medicinal value. EF is traditionally used in the treatment of headaches, abdominal pain, and menstrual pain. Moreover, studies [61] have found that Evodiamine (EVO) is one of the main bioactive ingredients contained in *Fructus officinalis*, which has potential anticancer effects. Its specific mechanism is to inhibit malignant tumors by inhibiting cell proliferation, metastasis, and invasion. Chen *et al*. [62] used network pharmacology and the QSAR model to study the anti-liver cancer effect of EF. 27 key compounds were obtained from two databases, TCMID and TCMSP, 45 intersection targets were obtained using the Venn Diagram, and SRC was identified as the main target of EF in the treatment of liver cancer using network integration analysis. The stable binding of SRC to the active compound was confirmed by molecular docking results, which further confirmed its anti-liver cancer properties. The model proposed in this study provides new theoretical support for further screening of effective TCM compounds against liver cancer.

#### 3.1.4 Deficiency Tonic Medicine

Positive deficiency is one of the main pathological features of the decline of zang-fu function in patients with liver cancer or liver cancer after surgery. Many doctors believe that the positive deficiency of liver cancer is nothing more than early physical weakness. Positive qi alone is not enough to ward against evil or suffer from turbid phlegm, nauseating heat, blood stasis, and other internal and external bad consumption harms to healthy Qi. Therefore, in the early or late stage of a disease, it is an important governance principle to pay attention to the use of the method of fuzheng to “first secure the place that is not affected by evil” or to cultivate and replenish the vitality, coordinate Yin and Yang, and resist evil. As one of the widely used Chinese herbal medicines, *Astragalus* membranaceus has been identified with over 100 compounds, including saponins, flavonoids, amino acids, and polysaccharides, which are often used as anti-cancer agents, tonics, liver preservatives, immune stimulants and diuretics [63]. *Astragalus* Homogeneous has an excellent tonic effect and can also treat diseases such as anal prolapse and splanchnosis.Astragalus MPaceus primarily impacted the mineral resorptive pathway of liver cancer, according to Liu's research using transcriptomics and network pharmacology [64]. It may also directly downregulate MT1G through dmitin, encourage iron death of liver cancer cells, and further improve the prognosis of liver cancer.

*Ginseng*, as one of the TCMs, has a good effect on tonic deficiency and is suitable for physical weakness and other diseases. The use of *ginseng* in TCM can be traced back to about 5, 000 years ago. Due to its advantages in pharmacokinetics, there are more and more studies on *ginseng* nowadays. Studies [65] have found that taking *ginseng* has good effects on human metabolism, the central nervous system, and even tumor diseases. Through bioinformatics and network pharmacology analysis, Chen *et al*. [66] found that there were 142 potential targets of Ginsenoside Rk1 and Rg5, the main active ingredients of *ginseng*, and 44 intersection targets related to liver cancer. Finally, it was found that G-Rk1 and G-Rg5 promote the endogenous apoptotic pathway of MHCC-97H cells by regulating key HCC-related genes and play a role in alleviating HCC by participating in the signaling pathway related to cell survival and proliferation.

#### 3.1.5 Laxative Medicine

Liver cancer is a malignant disease caused by external evil and internal injury. Therefore, in terms of treatment, catharsis is one of the main treatment methods. Dahuang has a good laxative effect, which is widely recognized by patients. Dahuang is also one of the traditional antitumor medicines commonly used in China and is often used in the treatment of liver cancer in clinics. Yu [67] used network pharmacology, molecular docking, and survival analysis to study the therapeutic effect of Dahuang on liver cancer, and constructed the overall network diagram of Dahuang anti-liver cancer medicine - ingredient - target - disease. He found that β-sitosterol and aloe-emodin are the two most important active ingredients in the treatment of liver cancer and play a role in the treatment of liver cancer through multiple signaling pathways such as cancer. Emodin is one of the important ingredients of Dahuang. Zhou *et al*. [68] found that 25 key genes of Emodin in the treatment of liver cancer are mainly enriched in the inflammatory response, neuropeptide signaling pathway and positive regulation of cytoplasmic calcium ion concentration, *etc*. In addition, GPR68, SSTR5, C5, LPAR6, P2RY4, and other targets may participate in the molecular mechanism of Emodin in the treatment of liver cancer and finally exert the effect of inhibiting liver cancer. It can be seen that Dahuang plays a significant role in the treatment of liver cancer, and its specific mechanism should be observed through more experiments in the future.

#### 3.1.6 Damp-clearing Medicine

The medicine of water infiltration and dampness is sweet and light, and the effect tends to be downward. It has the effect of water detumescence and diuresis. Modern pharmacological studies have proved that most medicines have diuretic, anti-tumor, liver protection, antihypertensive and other effects to varying degrees, and some medicines also have the effect of reducing blood sugar and regulating immune function. *Poria Cocos* as the representative of moistening medicine, has more than 2000 years of history, mainly distributed in China, America, Oceania, and Japan and Southeast Asia some countries. Previous pharmacological studies have found that polysaccharide is the most abundant substance in TCM *Poria Cocos*, and has a wide range of biological activities, including anti-tumor, anti-inflammatory, immunomodulatory, anti-hepatitis, anti-aging, anti-oxidation, *etc*., among which the anti-tumor property is particularly prominent, and it is mostly used in cancer patients during radiotherapy or chemotherapy [69]. As one of the important ingredients of *poria cocos*, Pachyman has been widely considered by many scholars. Qin has found through pharmacological studies that the key targets of Pachyman in the treatment of liver cancer include TNF, VEGFA, CASP3, ALB, SRC, *etc*. Molecular docking results showed that VEGFA and ALB may be the key targets of Pachyman in the treatment of liver cancer. Experimental studies have also confirmed that *in vitro* experiments after Pachyman treatment showed that the cellular content of VEGFA protein decreased while the intracellular level of ALB protein increased, ultimately confirming the positive effect of Pachyman on liver cancer [70].

#### 3.1.7 Astringent Medicine

Astringent medicine tastes more sour astringent, warm or flat and is mainly used for chronic physical deficiency; healthy qi is not solid; viscera function decline caused by sweating, night sweat, spermatospermia, spermatospermia, enuresis and other diseases of slippage. *Fructus Chebulae* is an herb widely used in traditional Asian medicine as an effective treatment for chronic dysentery and hoarseness.

Ellipticine and ellagic acid, as key extracts of *Fructus Chebulae*, have been proven to have anti-tumor activity. Jiang *et al*. [71] found through enrichment analysis that key genes of *Fructus Chebulae* in the treatment of liver cancer are mainly concentrated in the Ras signaling pathway, cAMP signaling pathway and other signaling pathways related to tumor metabolism. Through the MTT test, it was found that after treatment, the proliferation of HCC cells was significantly inhibited.

#### 3.1.8 Wind Quenching Medicine

Wind-quenching medicines are closely related to the liver. Most of the medicines are heavy and cold. They have the functions of calming the mind, clearing the liver and, improving the eyes and cooling the blood. Modern pharmacological studies have also found that most medicines have antihypertensive, sedative and anticonvulsive effects, and some medicines also have antipyretic and analgesic effects. As one of these TCMs, *Lily Reed* is often used to treat epilepsy, malaria, bad sores and so on in TCM. It also enhances immunity. As one of its key ingredients, resveratrol has attracted wide attention. A study has found that oxyresveratrol is a functional compound modified from the structure of resveratrol, which has significant antitumor effects [72]. Through experimental verification, Zhao *et al*. [73] found that clinical studies on liver cancer showed that ESR1 and EGFR mRNA expressions increased, while *in vitro* experiment data showed that the intracellular contents of ESR1 and EGFR mRNA decreased in liver cancer cells treated with oxyresveratrol. Therefore, the positive effect of oxyresveratrol on the treatment of liver cancer is illustrated.

## 4 MATCHED PAIR OF MEDICINE

Medicine pair, also known as medicine pair, was first seen in Beijing. It was a TCM based on the “Xiangxu Xiangshi” rational compatibility of TCM by doctors of all dynasties, which was achieved through summary and induction in clinical treatment. Two kinds of medicine can increase their overall therapeutic effect, which is the smallest unit of compatibility with TCM. The use of matched medicines in the treatment of liver cancer, more can play a unique therapeutic role. Ji *et al*. [74] found that the therapeutic effect of Qinghao-Kushen on HuH-7 and HepG2 cells was significantly better than that of single Qinghao or single Kushen, reflecting the advantages of pairing medicines. Further analysis showed that the treatment of HCC by Qinghao-Kushen mainly uses matrine and scopolamine and elucidates the inhibitory effect of key ingredients of Qinghao-Kushen on HCC cells.

*Radix Bupleuri*-*Rhizoma Cyperi* herb pair [75] is one of the commonly used treatments of liver cancer pairing medicines, the Qing through the enrichment analysis found that mainly through *Radix Bupleuri*-*Rhizoma Cyperi* herb pair p53 signaling pathway, metabolic pathways, and cell cycle, alleviate the occurrence of liver cancer development, Furthermore, the expression patterns, clinical features and prognosis of CDK1, MYC, CDK4, CCNB1, CDKN2A genes in cell cycle pathway were systematically analyzed using the Cancer Genome Atlas (TCGA) -HCC database, finally revealing the clinical value of *Radix Bupleuri*-*Rhizoma Cyperi* herb pair in inhibiting liver cancer. The results of molecular docking were verified.

## 5 CLINICAL EXPERIENCE PRESCRIPTION

Gu Pi Xiao Ji Prescription (GPXJP) is an empirical clinical prescription for the treatment of chronic liver disease. Yu *et al*. [76] found that it contains 171 active ingredients and 259 potential medicine targets through network pharmacology combined with *in vitro* experiments, and the specific mechanism of action involves 181 pathways *in vitro*. The final result confirmed that GPXJP can disrupt energy metabolism of liver cancer cells by regulating key gene fusion and mitochondrial division and ultimately play a role in the treatment of liver cancer. *In vitro* experiments, GPXJP can also inhibit the migration and proliferation of HepG2.2.15 cells by up-regulating MFN2 and MFN1 and ultimately down-regulating the expression of OPA1 and FIS1 and destroying mitochondria, thus achieving the therapeutic effect of liver cancer.

Bu Shen Jian Pi decoction (BSJPD) by Liu Wei Di Huang decoction (LWDHD) and Si Jun Zi decoction (SJZD), a merger of clinical experience formula, In order to explore the therapeutic mechanism of BSJPD for liver cancer, Wu *et al*. [77] conducted pharmacological studies *in vivo* and *in vitro*, among which nine key compounds were important nodes of BSJPD in the treatment of liver cancer, and the time results showed that the apoptosis rate of liver cancer cells in mice treated with BSJPD increased significantly. Finally, it was found that BSJPD prolonged the survival period of patients with liver cancer and further induced the apoptosis of tumor cells, which may be related to the regulation of the expression of p53, PI3K, CASP3, Akt, *etc*.

Yi Qi Jian Pi Jie Du(YQJPJD) formula is also one of the clinical experience prescriptions for the treatment of liver diseases, and the effect is very significant. Wu [78] found through network pharmacological analysis that the YQJPJD formula mainly regulates potential therapeutic targets of liver cancer through key ingredients such as luteolin, quercetin and baicalin and exerts anti-liver cancer effects through core targets such as RAC1, MAPK3, and RHOA. Meanwhile, experimental research found that the YQJPJD formula can significantly inhibit the invasion, proliferation and migration of liver cancer cells and promote the apoptosis of liver cancer cells.

San Shi Mao (SSM) formula is a clinically effective formula based on the theory of promoting blood circulation and detoxification, which has a good effect on liver cancer. Yu *et al*. [79] obtained through molecular biology and network pharmacology analysis that the core targets of SSM formula in the treatment of liver cancer included MAPK1, JUN, HSP90AA1, TP53, EGFR, and AR, and after SSM treatment, the vitality and migration of liver cancer cells were reduced, and apoptosis was further promoted. The EGFR/FAK/AKT signaling pathway is effectively inhibited and finally plays a role in the treatment of liver cancer.

Ruan Gan Li Dan decoction (RGLD), by the name of the formula, can be found on the main established for liver disease and summarizes the clinical experience of prescriptions, especially for the treatment of liver cancer. Chen [80] *et al*. found 25 key targets of RGLD in the treatment of liver cancer and further constructed the RGLD compose-target network diagram formed by 216 ingredients and 306 targets. Through multi-level systematic pharmacological analysis, they found that the specific mechanism of RGLD in the treatment of liver cancer involved cell proliferation and cell response to hypoxia, *etc*. Moreover, it can inhibit liver cancer through the PI3K/Akt signaling pathway, MAPK signaling pathway and TNF signaling pathway.

## 6 TRADITIONAL CHINESE MEDICINE COMPOUND 

TCM is rich in a variety of active ingredients, especially TCM compounds, which have the advantages of multi-pathway and multi-target, which can avoid adverse therapeutic effects caused by genetic defects of chemical therapeutic targets and weakened medicine effects caused by genetic defects of medicine metabolism. At present, a large number of research results have shown that TCM compounds have anti-tumor, anti-oxidation, anti-inflammation, anti-fibrosis, anti-apoptosis and other effects. In the treatment and prevention of liver cancer, these biological pathways are interrelated and play a role in a multi-ingredient, multi-target and multi-pathway way. Xuan Fu - Hua decoction of TCM compound for the treatment of polio, in view of the “liver blood stasis” related disease, curative effect is very obvious, such as liver, fatty liver, cirrhosis of the liver, cholecystitis, *etc* [81, 82]. A method based on the transcriptome study and network pharmacology, some scholars [83] found Xuan Fu - Hua decoction can significantly increase the 5-fluorouracil (5-FU) induced liver cancer SMMC - 7721 cells *in vivo* and *in vitro* growth inhibition and apoptosis and significantly improve the ZMAT3, PMAIP1 and DDIT3 gene transcription. Transcription levels of GFBP3, TGFBR1, NFE2L2, AXIN2, MITFI and WNT4 genes were reduced. At the same time, Gene set enrichment analysis also showed. Eventually, Xuan Fu - Hua decoction is enhanced by inhibiting cell survival and cell apoptosis mechanism to increase the liver cancer cells for the treatment of 5-FU cytotoxic sensitivity.

Yin Chen Hao Decoction (YCHD) was first recorded in the Han Dynasty. Famous doctor Zhang Zhongjing's theory of typhoid miscellaneous disease consists of gall, rhubarb, gardenia, has clear heat dehumidification, the effect of cholagogue retreat yellow, is the first main prescription of TCM treatment of damp heat jaundice. Medical studies have shown that Yinchenhao Decoction has good clinical efficacy in the treatment of liver cancer, alcoholic liver disease, cholestatic jaundice, acute jaundice hepatitis, and lowering blood lipid and blood sugar. In order to explore the main mechanism of action of YCHD inhibiting liver cancer, Sun *et al*. [84] used network pharmacology to conduct research and found that YCHD directly affected 17 disease targets related to liver cancer and finally speculated that YCHD played a role in inhibiting liver cancer through metabolic pathways and regulating inflammation.

Xihuang pill has been widely used in the treatment of cancer patients as a TCM compound to inhibit the proliferation of tumor cells [85]. Relevant studies [86] have shown that the Xihuang pill can inhibit tumor growth, proliferation, and metastasis by regulating the JNK1/AP-1 signaling pathway, PI3K/AKT signaling pathway or MEKK1/SEK1 signaling pathway. Zhao *et al*. [87] established a network of medicine constituents and targets and found that there were 106 overlapping genes between molecular targets and medicine targets of liver cancer, and finally identified VEGFA and EGFR as potential therapeutic targets of Xihuang pill for liver cancer. Another network pharmacological analysis [88] identified quercetin as the key active ingredient in the Xihuang pill by constructing the ingredient -- disease -- target network diagram and found that quercetin could significantly inhibit the migration, proliferation and invasion of liver cancer cells and effectively promote cell apoptosis through *in vitro* experiments. Finally, the NF-κB signaling pathway promotes autophagy and regulates macrophage polarization.

Da Huang Zhe Chong pill (DHZCP) was first recorded in Jingui Yaolue by Zhang Zhongjing, a physician of the Han Dynasty. It is often widely used in the treatment of various liver diseases. However, its clinical application is still limited due to the combination of severe medicines such as rhubarb and blood-breaking and blood-silting insect medicines. Chen's study [89] explored the mechanism of DHZCP enhancing anti-tumor immunity by using an in situ liver cancer model in mice. After DHZCP treatment, Th1 cells in the spleen and peripheral blood of mice increased and secreted more IFN-γ, thus activating CD8+T cells, further inhibiting the generation of Treg cells, and finally inhibiting the growth of liver cancer cells.

Ge Xia Zhu Yu Decoction (GXZY) is a classical prescription for TCM medicinal plants. Cao *et al.* [90] analysis showed that the core genes predicted by GXZY on liquid chromatography were MYC, JUN, AR, MMP9, CASP3, RELA, *etc*., which inhibited liver cirrhosis by regulating hepatitis B, cancer, MAPK signaling pathway, and TNF signaling pathway, and alleviated the progression to liver cancer. *In vivo*, experiments confirmed that GXZY regulates cell migration and proliferation mainly by reducing the expression of MMP9.

Huang Lian Jie Du decoction (HLJDD) is a classic square that is used for heat-clearing and detoxifying. It is composed of Coptidis, scutellaria, Phellodendri and gardenia. Its chemical ingredients are complex and diverse, leading to its wide pharmacological effects. Another network pharmacological analysis [91] showed that cancer, especially liver cancer, ranked first in the predicted diseases treated by HLJDD. However, genes related to inflammation play an important role in the treatment of cancer by HLJDD. Then, the significant inhibitory effect of HLJDD on liver cancer *in vivo* was further demonstrated by transplantation of liver cancer in situ into mouse models.

## 7 CHINESE PATENT MEDICINE

Nowadays, the application of Chinese patent medicine to treat liver cancer is also very common. Compound Kushen Injection (CKI) is a common antitumor injection used in the treatment of liver cancer [92-94]. Lu *et al*. [95] concluded through network analysis that CDK1, BCHE, ADH1A, ADH1C, EPHX2, and SRD5A2 were the core targets of CKI inhibiting liver cancer.It was discovered through *in vitro* research that CKI could effectively treat liver cancer by greatly inhibiting the proliferation of HepG5 cells, up-regulating ADH1A and SRD5A2, and down-regulating EPHX2 and CDK2.

Compound mylabris capsules (CMC) are also a classic Chinese medicine formula, which is made into Chinese patent medicine with obvious clinical efficacy. Wei *et al.* [93] analyzed and found that there were 34 overlapping genes between CMC and HCC. *In vitro* studies showed that the medicine serum of CMC could significantly inhibit the viability of HepG2 cells. CMC can also down-regulate the expressions of CDK1 and CCNB1, induce G2/M phase cell cycle arrest, and play a role in the treatment of liver cancer through a multi-ingredient, multi-target, and multi-pathway mechanism.

KangXianYiAi (KXYA) formula granules are often used in the clinical treatment of liver disease. Cao [96, 97] found that The granule of the KXYA formula treated HBV-related liver cancer by regulating the expression of genes such as MAPK8, HDAC1, PTEN, NR3C1, EGFR and HNF4A. The major pathways involved include cancer, hepatitis B, and PI3K-Akt signaling pathway, which ultimately inhibit the growth of liver nodular in patients with chronic hepatitis B, impede the migration and proliferation ability of tumor cells, and promote the apoptosis of HepAD38 cells.

Huachansu injection is widely used in the treatment of various solid tumors such as HCC, gastric cancer, gallbladder cancer and advanced non-small cell lung cancer in China [98]. Through comprehensive bioinformatic analysis, Huang *et al*. [99] found that TTK, AURKB, MMP12, AKR1B1, CDK1, CHEK1 and AURKA may be potential up-regulated target proteins of Huachansu injection in the treatment of liver cancer. The mechanism of Huachansu injection in the treatment of liver cancer by these up-regulated targets is related to the p53 signaling pathway, viral carcinogenesis, cell senescence and cell cycle. It was also found that NR1I2, SRD5A2, and SHBG may be potential down-regulated target proteins of Huachansu injection in the treatment of liver cancer. The results of molecular docking confirmed this view. All the above studies have confirmed the significant therapeutic effect of some Chinese patent medicines on liver cancer, and the application of network pharmacology once again reflects the characteristics of multi-ingredient, multi-target and multi-pathway treatment of TCM.

## CONCLUSION

We have made some progress in the inheritance and innovation of TCM in the prevention and treatment of HCC, especially in the inhibition of tumor cell proliferation, regulation of the immune microenvironment, anti-metastasis, induction of apoptosis, and improvement of patient's quality of life, *etc*., and the difficulties and entry points of TCM in the treatment of HCC are the hotspots of current research. Nowadays, the combination of TCM and network pharmacology is an important direction of modern Chinese medicine research, which utilizes modern scientific and technological means, especially big data analysis [100] and artificial intelligence [101], so as to reveal the complex mechanism of action and the material basis of the medicinal effect of TCM in a deeper way. This combination not only contributes to the internationalization and modernization of TCM but also promotes precision therapy and the discovery of new medicines in TCM.The use of network pharmacology to conduct comprehensive research on the precise mechanism of action can finally successfully promote the inheritance and development of TCM while fully utilizing the benefits of the entire diagnosis and treatment of TCM.

The research strategy of network pharmacology on TCM is in line with the understanding of TCM on the nature of diseases, and it is clear that TCM compound is a system of multi-ingredient synergistic action. By acting on multiple targets of different organs, it regulates multiple biological processes and functions, thus giving full play to the overall therapeutic mechanism and providing a new idea for solving the problems of unclear effective ingredients and mechanism of action of TCM. As a new method of TCM research, network pharmacology research cannot be separated from a variety of databases and technical means. At present, there are still some problems to be solved, such as incomplete data in the database, a limited number of medicine targets, and small molecular compounds obtained by text mining, which cannot fully reveal their pharmacological effects. The network model based on data also makes it difficult to reflect on the overall situation and has certain limitations. At present, the network pharmacology of TCM is in the initial stage of research and needs further innovation and development in research strategies, methods, and key technologies. It is believed that in the near future, TCM research based on network pharmacology will deepen people's understanding of the mechanism of TCM in the treatment of liver cancer, promote the inheritance and innovation of TCM, and accelerate the process of TCM modernization and internationalization.

## Figures and Tables

**Table 1:** Public databases related to TCM network pharmacology.

**Type**		**Name**	**Description**	**Website for Database or Tool**	**References**
Databases	Database of disease target screening	DrugBank	A database for retrieving information on drugs and drug targets, known as an encyclopedia of drugs	https://go.drugbank.com/	Wishart, *et al.* (2018) [19]
-	-	OMIM	A database containing information on more than 16, 000 genes	https://www.omim.org/	Amberger, *et al.* (2015) [20]
-	-	GeneCards	A comprehensive database of transcriptional, proteomic, genomic and protein targets for clinical diseases	https://www.genecards.org/	Stelzer, *et al.* (2016) [21]
-	-	PharmGkb	An authoritative database dedicated to pharmacogenomics	https://www.pharmgkb.org/	Gong, *et al.* (2021) [22]
-	-	TTD	A free database that provides information on the main targets of the medicine	http://db.idrblab.net/ttd/	Zhou, *et al.* (2022) [23]
-	Database for screening of active ingredients and target prediction	SwissTarget Prediction	A database capable of predicting possible small molecule target proteins by reverse screening	http://swisstargetprediction.ch/	Daina, *et al.* (2019) [24]
-	-	TCMSP	A commonly used database for screening medicine ingredients and targets using network pharmacology methods	https://old.tcmsp-e.com/tcmsp.php	Ru, *et al.* (2014) [25]
-	-	TCMID	A powerful TCM database integrating medicine, disease, target, and prescription research	http://bidd.group/TCMID/index.html	Ji, *et al.* (2006) [26]
-	-	Pharmmapper	A platform for pharmacophore matching and potential target identification	http://lilab-ecust.cn/pharmmapper/index.html	Wang, *et al.* (2017) [27]
-	-	BAT-MAN	An online bioinformatics analysis tool specifically designed for studying the molecular mechanisms of traditional Chinese medicine	http://bionet.ncpsb.org.cn/batman-tcm/	Liu, *et al.* (2016) [28]
-	PPI Network and Database for Bioenrichment Analysis	STRING	A database of known or predicted interactions between proteins	https://cn.string-db.org/	Szklarczyk, *et al.* (2021) [28]
-	-	Metascape	An easy-to-use and powerful tool for gene function annotation analysis	https://metascape.org/	Zhou, *et al.* (2019) [30]
-	-	DAVID	A bioinformatics database designed to provide systematically synthesized annotated information on biological functions for large-scale gene or protein lists	https://david.ncifcrf.gov/	Dennis, *et al.* (2003) [31]

## LIST OF ABBREVIATIONS

5-FU: 5-fluorouracil
ACDPs: A.cinnamomea dropping pills
BSJPD: Bu Shen Jian Pi decoction
CKI: Compound Kushen Injection
CMC: Compound mylabris capsules
CPT: Cryptotanshinone
DHZCP: Da Huang Zhe Chong Pill
EF: Evodiae Fructus
EVO: Evodiamine
GPXJP: Gu Pi Xiao Ji Prescription
GXZY: Ge Xia Zhu Yu Decoction
HBV: Hepatitis B virus
HCV: Hepatitis C virus
HDW: Hedyotis difusa Willd
HLJDD: Huanglian Jiedu decoction
KXYA: KangXianYiAi
LWDHD: Liu Wei Di Huang decoction
PPI: Protein-protein interaction
RGLD: Ruan Gan Li Dan decoction
SJZD: Si Jun Zi decoction
SSM: San Shi Mao
SVR: Sustained virologic response
TanIIA: Tanshinone IIA
TCGA: The Cancer Genome Atlas
TCM: Traditional Chinese medicine
YCHD: Yin Chen Hao Decoction
YQJPJD: Yi Qi Jian Pi Jie Du
